# Using balance training to improve the performance of youth basketball players

**DOI:** 10.1007/s11332-013-0143-z

**Published:** 2013-04-30

**Authors:** Gabriele Boccolini, Alessandro Brazzit, Luca Bonfanti, Giampietro Alberti

**Affiliations:** Department of Biomedical Sciences for Health, Faculty of Excercise Sciences, Università degli Studi di Milano, Italy, Via G. Colombo, 71, 20133 Milan, Italy

**Keywords:** Proprioception, Stability, Vertical jump, Basket

## Abstract

The aim of this study was to evaluate the effectiveness of 12 weeks of balance training to improve the balance and vertical jump abilities of young basketball players. Twenty-three players from two teams in the Under Fifteen Basketball Excellence category participated in the study. Participants were divided into two training groups: balance training (BAL, *n* = 11) and isotonic training (ISO, *n* = 12). Both groups were tested for balance and vertical jumps at the beginning of the competitive season and at the end of 12 weeks of specific training programme. All of the tests were performed in sustained bipodalic and monopodalic (both right and left) positions. The results showed that players who participated in balance training for 12 weeks, compared to players who trained with isotonic machines, exhibited a significantly increase in balance (bipodalic 28.3 %; right 41.4 %; left 45.8 %; *p* < 0.01) and muscular power (bipodalic 8.1 %; right 13.5 %; left 12.5 %; *p* < 0.01) as measured through a vertical jump. In conclusion, balance training using unstable boards was an effective training method for improving balance and the vertical jump, which is a basketball-specific action that frequently occurs in this sport.

## Introduction

Balance training is an interesting and controversial method of training for coaches because of the transversal effect that it may have on athletic performance in various sports and at different ages. From the literature, an evidence-based relationship between balance ability and the risk of injury is clear. In fact, studies have demonstrated that systematic training in a balance training program would be effective in reducing the risk of injuries [1, 2]. It was observed that when balance training was implemented during competitive season, the occurrence of injury rate was reduced by 38 % [3].

However, the link between balance ability and athletic performance is not fully clear and required further evidence. There are, in fact, very few studies that specifically investigated balance training as approach to improving performance [4, 5].

Because of growing scientific knowledge, the role of neuromuscular training has become better known [6]. Moreover, it has been used in combination with balance training in different sports, such as basketball, soccer and gymnastics [4].

Balance can be defined as the ability to maintain the body’s centre of gravity over the base of support and results from neuromuscular actions in response to continuous visual, vestibular and somato-sensory feedback.

In recent years, balance training has become a very popular addition to more standard athletic training programme in many sports. Basketball, for example, requires the players to habitually address physical contact and various situations involving balance instability, such as basketball-specific accelerations and decelerations, changes in direction, penetrations into the defensive perimeter, boxing out, dribbling and defence position recovery. These actions are often performed in a very limited space and require very fast movement, high coordination ability and appropriate strength.

Disequilibrium can be found in every specific movement of basketball, such as in the twisting movement of feet (particularly the pivot foot), jump shots as well as offensive and defensive rebounds. Hence, further investigation of the most suitable balance training protocols for basketball is warranted.

In basketball, the standard strength takes a leading role in training; strength training allows players to improve their physical abilities and athletic performance and, therefore, their outcomes during games and to prevent injuries.

In particular, the vertical jump is one of the most frequently repeated actions. Among vertical jumps, the countermovement jump (CMJ) is skill in basketball, and it is also the technique most frequently used as an evaluation test of the physical condition of the lower limbs [7].

It is important to note that several studies have demonstrated that balance training can be used to improve performance related to postural control as well as to improve physical and athletic activities such as the time on the ball, the shuttle run and the vertical jump [8]. A neuromuscular training program that is focused on core stability, specifically the lower extremity strength, would affect the performance of an athlete in the Star Excursion Balance Test [6]. A study on collegiate women athletes showed that 10 min of unstable surface training 4 days a week would improve the single-leg squat and the 1-min sit-up test performance [9]. Further studies compared the effects of balance training to other strength training methods [5]. Among them, the effect of plyometric training on muscular power, strength, balance and landing force has been compared to the effect of balance training, suggesting that both plyomentric and balance training increase neuromuscular power and control [10].

Furthermore, in [11], which included athletes of different sports, balance training was demonstrated to reduce muscular strength imbalances between the legs and to be effective for gaining muscular strength [11].

Thus, the aim of this study was to compare the effect of 12 weeks of balance training to 12 weeks of traditional strength training using leg press and leg extension isotonic machines in young basketball players. To do so, this study evaluated the training-induced effects on balance and vertical jump outcomes, which is a basketball-specific action.

## Methods

### Design

In the present study, a longitudinal, parallel and controlled design was used. The subjects were randomly allocated into the balance group (BAL, *n* = 11), which performed a balance training intervention twice a week for 30 min for 12 weeks, and the isotonic group (ISO, *n* = 12), which performed traditional strength training with isotonic machines (i.e., the leg extension and leg press) twice a week for 30 min for 12 weeks.

### Subjects

Twenty-three young male basketball players of two different Under Fifteen Basketball Excellence teams participated in this study (participants characteristics are given in Table 1). Before participation, subjects and parents were informed about the aim and the design of the study, and they signed a written informed consent. Only subjects who did not report any injuries in the 6 months prior to the study were recruited.

**Table 1:** Overview of participant characteristics (mean ± SEM)

	BAL group	ISO group
Age (years)	15.0 ± 0.0	14.6 ± 0.1
Weight (kg)	69.6 ± 3.0	65.5 ± 1.7
Height (kg)	180.1 ± 2.4	178.6 ± 2.6
BMI (kg m^−2^)	21.3 ± 0.7	20.6 ± 0.3

### General procedures

All the subjects began the pre-season period in September, and this study began in October at the beginning of the in-season period. Pre- and post-testing was performed on Tuesday starting at 4:00 p.m., which was not close to the game days. The games were played on Sunday. Anthropometric data were measured before testing.

### Pre–post-testing

Twenty-three subjects were evaluated on balance ability using a balance test (Libra board, Easytech, Prato, Italy) and on vertical jump ability using a CMJ Test (Optojump, Microgate, Bolzano, Italy). At the end of each specific training intervention, post-testing occurred 12 weeks after pre-testing. In post-testing, the same procedures and sequences were used as in the pre-testing.

After a revision of the test protocols and the survey procedures, subjects participated in a standard warm-up consisting of 5 min of jogging at a comfortable speed followed by 2 min of static stretching of the lower extremity muscles. This warm-up procedure was selected to avoid any variation in the usual warm-up of the players. A balance test was performed first, while the countermovement jump test was performed after. Sustained bipodalic and monopodalic (both left and right) positions were evaluated.

#### Balance test

Balance ability was evaluated through the balance test using the Libra board (Easytech, Prato, Italy). This instrument (42 × 42 cm, weight 2.7 kg) consists of a balancing board with a wide antislip area. A Libra board is connected to a personal computer, and balance ability can be assessed with appropriate software (Libra software, version 2.2). The wide support area of the Libra board consists of three interchangeable plugs, making it possible to have different degrees of structural difficulty (40 cm = high; 24 cm = moderate; 12 cm = easy). Subjects were asked to fixate on a point on the wall, in the eye plane, at a distance of 3 m. A moderate degree of difficulty was set, with the board plug placed at 24 cm.

The balance test was performed using the Libra board on the lateral plane in three stance positions, including bipodalic and monopodalic (both left and right) positions. Three trials per position were executed, with each position held for 30 s and a rest of 30 s between successive trials. The mean result of the three trials was used as the test outcome. The Libra board software returns the outcomes in arbitrary units. The scores range between 0 and 100, and the better results are closer to 0. The same instrument and the same protocol have been used in other studies [12–14].

#### Countermovement jump test

The muscular power of the lower limbs was assessed using Bosco’s procedures [15], specifically the countermovement jump test (CMJ) with a free arm-swing, which is performed under three different conditions, namely, bipodalic and monopodalic (both left and right) positions. Subjects started from an upright standing position, and they were instructed to flex their knees (approximately at 90°) as quickly as possible and then to perform a vertical jump as high as possible, with a free arm-swing. The test was performed using the Optojump system (Microgate, Bolzano, Italy), an optical infrared validated device that can assess the height of a vertical jump [16]. The height of the CMJ is indirectly estimated as the following: 9.81 × (flight time)^2^ × 8^−1^ [15]. The height of the vertical jump correlates with the power output of the leg extensor muscles during natural motion [15, 17], with the 1 RM in half-squat and 10 m shuttle test performance [18].

In the pre-test condition analysis, no significant differences were found between the BAL and ISO groups (*p* > 0.05) in terms of their anthropometric characteristics and the balance and CMJ tests. All of the subjects were assumed to be in the same pre-intervention condition.

### Training intervention

The BAL and ISO groups started the training protocol on the same day; it was performed for 12 consecutive weeks. Both the BAL and ISO groups performed the specific training on Tuesdays and Fridays (Table 2). Each specific training session lasted approximately 30 min. Before starting the training session, subjects completed their usual warm-up. The warm-up was composed of three phases: (1) 5 min of jogging at a comfortable speed; (2) 2 min of static stretching for the lower limb muscles; and (3) 5 min of shooting, from both sides of the court.

**Table 2:** Overview of the weekly training microcycle for the BAL and ISO groups

Tuesday	Wednesday	Friday	Sunday
30 min balance/isotonic training 90 min basket	90 min basket	30 min balance/isotonic training 90 min basket	League game

ISO group conducted a specific training session using the leg press and leg extension isotonic machines (Technogym Inc.). Each ISO subject performed the following: 5 sets of 12 leg press repetitions at 70 % 1 RM with 3 min of recovery and 4 sets of 10 leg extension repetitions at 70 % 1 RM with 3 min of recovery. This training regime was primarily focused on increasing the strength of the lower limbs. The strength training protocols were in accordance with the ACSM’s 2009 position stand on resistance training. ACSM recommended that novice to intermediate individuals train with loads corresponding to 60–70 % of 1 RM for 8–12 repetitions [19].

The main purpose of the specific balance training was to improve balance ability through training with unstable surfaces, such as the Swiss-Ball (Perform Better Inc.) and the Trial-T1 half-sphere (TRIAL s.r.l., Forlì, Italy, 45 × 23 cm). The following balance training was performed by all of the subjects. First, subjects performed 8 sets of 20 s of Swiss-Ball kneeling hold balancing with 30 s of recovery; subjects were told to maintain balance on the Swiss-Ball while keeping their knee at an approximate angle of 90°. Then, they performed 6 sets of 20 repetitions of the two-handed chest pass balance exercise with 30 s of recovery; subjects were instructed to maintain balance on the Trial-T1 half-sphere while performing 20 chest passes to a teammate in front of them at a distance of approximately 10 m. Finally, subjects performed 10 sets of 30 s per limb of the single-leg balance, with 10 s of recovery between repetitions and alternating the supporting lower limb. Subjects were instructed to maintain balance on the Trial-T1 half-sphere while keeping a knee angle of approximately 120°.

In addition to balance or isotonic training, both the BAL group and the ISO group completed 3 technical-tactical training sessions with their head coach, two of which occurred at the end of specific training, for a total time of approximately 4 h and 30 min per week. The athletes’ weekly schedule is shown in Table 2.

### Statistical analyses

Statistical analyses were performed with SPSS software (version 13.0; SPSS Inc., Chicago, IL, USA). All data are presented as the mean ± standard error of the mean (SEM). To check the reliability of the balance and CMJ tests, the intraclass correlation index (ICC) was applied [20] with 95 % confidence intervals (CI) and coefficients of variation (CVs). ICC shows an acceptable reliability for all of the tests (Table 3).

**Table 3:** Reliability of the balance and CMJ tests

	ICC (95 % CI)	CV (%)
Balance—bipodalic (AU)	0.90 (0.86–0.95)	1.5
Balance—right (AU)	0.88 (0.86–0.91)	1.7
Balance—left (AU)	0.90 (0.88–0.92)	1.3
CMJ—bipodalic (cm)	0.98 (0.97–0.98)	1.7
CMJ—right (cm)	0.94 (0.93–0.95)	1.9
CMJ—left (cm)	0.96 (0.96–0.98)	2.0

A paired *t* test was used to evaluate the differences between the pre- and post-tests, while an unpaired *t* test was used to compare the pre-test conditions of the BAL and ISO groups. The significance was set at an alpha level of 0.05.

## Results

**Fig. 1:**
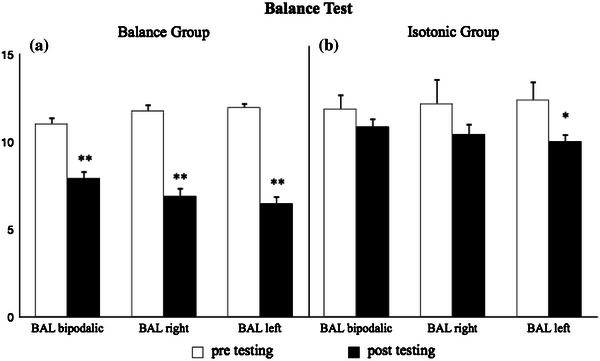
Balance ability of the BAL group (**a**) and of the ISO group (**b**) before and after 12 weeks of balance training and of strength training with leg press and leg extension isotonic machines, respectively, assessed by the balance test in sustained bipodalic and monopodalic (both *right* and *left*) positions. **p < 0.01 *p < 0.05

**Fig. 2:**
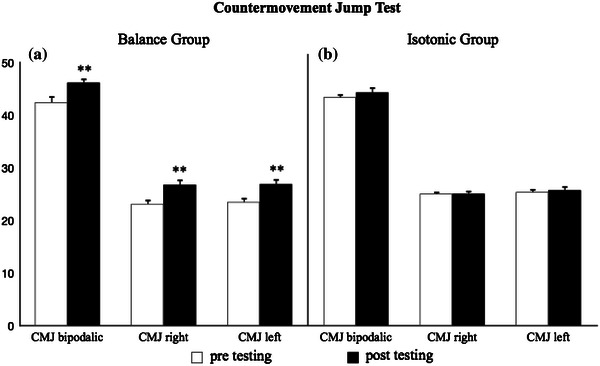
CMJ height for the BAL group (**a**) and for the ISO group (**b**) before and after 12 weeks of balance training and of strength training with leg press and leg extension isotonic machines, respectively, assessed by the CMJ test in bipodalic and monopodalic (both *right* and *left*) positions. **p < 0.01

The results of the two groups after 12 weeks of specific training were evaluated. The pre- and post-test results are shown in Figs. 1 and 2.

Following 12 weeks of balance training, the BAL group showed significant improvement (*p* < 0.01) in both the balance (bipodalic 28.3 %; right 41.4 %; left 45.8 %) and CMJ tests (bipodalic 8.1 %; right 13.5 %; left 12.5 %) (Figs. 1a, 2a).

Following training with the isotonic machines, the ISO group showed significant improvement (*p* < 0.05) only in the left monopodalic balance test (left: 16.39 %) (Fig. 1b).

## Discussion

The aim of the present study was to evaluate the effectiveness of 12 weeks of balance training to improve the balance and vertical jump abilities of young basketball players.

Twelve weeks of balance training allowed the BAL group to significantly improve their performance (*p* < 0.01, Figs. 1a, 2a) on all of balance and vertical jump tests, while 12 weeks of strength training with leg press and leg extension machines resulted in a significant improvement (*p* < 0.05, Fig. 1b) for the ISO group only in the left leg balance test. Both the BAL and ISO groups were randomly selected at the beginning of the regular season; therefore, the players had already completed the pre-season training with their head coach. This scenario ensured that all of the participants started the specific training protocols of the study in equal physical and athletic conditions.

The significant improvements that the BAL group achieved in the vertical jump (Fig. 2a) emphasise a higher muscular strength outcome than at pre-testing, as expressed in natural actions such as jumps. That could depend on improvement in the intra- and inter-muscular coordination of the knee extensors.

The findings of our study agree with other similar studies. In fact, significant improvements in the vertical jump abilities have been found in similar studies on balance training. Kean et al. [21], Myer et al. [10] and Taube et al. [17] examined recreationally active females, young females practicing different sports and winter sports athletes, respectively, and all three of these studies obtained the same results with respect to jump abilities [10, 17, 21]. However, the main difference between the aforementioned and our investigation involves the period during which these studies were performed. In Taube et al. [17], balance training was performed at the beginning of the pre-season training, whereas in Myer et al. [10], it was performed during an off-season period.

Thus, some of the differences between our and previous studies may be given to the study’s particular period of implementation, the sport and the participants.

Compared to the BAL group, the ISO group did not show a significant improvement in the vertical jump (Fig. 2b). In contrast to our results, Heitkamp et al. [11] found some muscular strength improvement in two groups that underwent, respectively, balance training and strength training with leg curl and leg press machines. However, in that study, muscular strength was evaluated on the Cybex Norm Isometric System to derive the isometric maximum strength; whereas in our study, the height of the vertical jump was evaluated. Therefore, these differences could result from the fact that the actions performed on the Cybex would be similar to the actions performed on isotonic machines. This represents a different scenario from the vertical jump because of the reuse of the elastic component that occurs in the specific action.

Referring to balance ability, the BAL group showed a significant improvement (*p* < 0.01) in the sustained bipodalic and monopodalic positions (Fig. 1a).

These results are in accordance with the findings of several other studies [6, 10, 11, 17, 21, 22].

Thus given the young age of our subjects, balance training appears to be a notable method for improving the vertical jump.

The most interesting and unexpected results concern the significant improvement (*p* < 0.05) that the ISO group attained in the left leg monopodalic balance test (Fig. 1b). We can speculate that this difference may result from the fact that players used the left foot for takeoff as the preferred foot in layups. The repetition of this action under unstable conditions, as usually happens in the challenges during the match, might have generated some proprioceptive mechanisms, inducing the ISO group to improve only its left leg balance ability.

## Conclusion

The results of this study show that performing 30 min of balance training twice a week for 12 weeks induces a significant improvement in balance and vertical jump scores, both in sustained bipodalic and in monopodalic positions. For this reason, aside from being a valid training method to prevent basketball injuries, balance training using unstable boards is an effective training method for improving balance and the vertical jump, which is a basketball-specific action that frequently occurs in that sport. This may especially be the case when balance training is performed by young amateur athletes. This scenario is a very inexpensive and efficacious training method that every basketball coach should consider.

## References

[1] Hubscher M, Zech A, Pfeifer K, Hansel F, Vogt L, Banzer W (2010). Neuromuscular training for sports injury prevention: a systematic review. Med Sci Sports Exerc.

[2] Zech A, Hubscher M, Vogt L, Banzer W, Hansel F, Pfeifer K (2009). Neuromuscular training for rehabilitation of sports injuries: a systematic review. Med Sci Sports Exerc.

[3] McGuine TA, Keene JS (2006). The effect of a balance training program on the risk of ankle sprains in high school athletes. Am J Sports Med.

[4] Hrysomallis C (2011) Balance ability and athletic performance. Sports Med (Auckland, NZ) 41:221–232 10.2165/11538560-000000000-00000 21395364

[5] Zech A, Hubscher M, Vogt L, Banzer W, Hansel F, Pfeifer K (2010). Balance training for neuromuscular control and performance enhancement: a systematic review. J Athl Train.

[6] Filipa A, Byrnes R, Paterno MV, Myer GD, Hewett TE (2010). Neuromuscular training improves performance on the star excursion balance test in young female athletes. J Orthop Sports Phys Ther.

[7] Ziv G, Lidor R (2010). Vertical jump in female and male basketball players–a review of observational and experimental studies. J Science Med Sport Sports Med Aust.

[8] Yaggie JA, Campbell BM (2006). Effects of balance training on selected skills. J Strength Cond Res Natl Strength Cond Assoc.

[9] Oliver GD, Di Brezzo R (2009). Functional balance training in collegiate women athletes. J Strength Cond Res Natl Strength Cond Assoc.

[10] Myer GD, Ford KR, Brent JL, Hewett TE (2006). The effects of plyometric vs. dynamic stabilization and balance training on power, balance, and landing force in female athletes. J Strength Cond Res Natl Strength Cond Assoc.

[11] Heitkamp HC, Horstmann T, Mayer F, Weller J, Dickhuth HH (2001). Gain in strength and muscular balance after balance training. Int J Sports Med.

[12] Bole R (1975). Relationship of objective score, perceptual trace, and practice method in learning to balance. Percept Mot Skills.

[13] Davlin CD (2004). Dynamic balance in high level athletes. Percept Mot Skills.

[14] De Gunsch E, Spielmann F, Van Meerhaege T, Di Palma E (2006) Contribution to the normalization of the balance board. In: XV congress on sport rehabilitation and traumatology. The rehabilitation of winter and mountain sports injuries

[15] Bosco C, Luhtanen P, Komi PV (1983). A simple method for measurement of mechanical power in jumping. Eur J Appl Physiol.

[16] Glatthorn JF, Gouge S, Nussbaumer S, Stauffacher S, Impellizzeri FM, Maffiuletti NA (2011). Validity and reliability of Optojump photoelectric cells for estimating vertical jump height. J Strength Cond Res Natl Strength Cond Assoc.

[17] Taube W, Kullmann N, Leukel C, Kurz O, Amtage F, Gollhofer A (2007). Differential reflex adaptations following sensorimotor and strength training in young elite athletes. Int J Sports Med.

[18] Wisloff U, Castagna C, Helgerud J, Jones R, Hoff J (2004). Strong correlation of maximal squat strength with sprint performance and vertical jump height in elite soccer players. Br J Sports Med.

[19] American College of Sports Medicine (2009) Progression models in resistance training for healthy adults. Med Sci Sports Exerc 41:687–708 10.1249/MSS.0b013e3181915670 19204579

[20] Atkinson G, Nevill AM (1998) Statistical methods for assessing measurement error (reliability) in variables relevant to sports medicine. Sports Med (Auckland, NZ) 26:217–238 10.2165/00007256-199826040-00002 9820922

[21] Kean C, Behm D, Young W (2006). Fixed foot balance training increases rectus femoris activation during landing and jump height in recreationally active women. J Sport Sci Med.

[22] Emery CA, Cassidy JD, Klassen TP, Rosychuk RJ, Rowe BH (2005). Effectiveness of a home-based balance-training program in reducing sports-related injuries among healthy adolescents: a cluster randomized controlled trial. CMAJ.

